# One-Stage Approach to Rehabilitate a Hopeless Tooth in the Maxilla by Means of Immediate Dentoalveolar Restoration: Surgical and Prosthetic Considerations

**DOI:** 10.1155/2024/5862595

**Published:** 2024-02-10

**Authors:** Gabriel Mulinari-Santos, Fabio Luiz Ferreira Scannavino, Erica Dorigatti de Avila, Luiz Antonio Borelli Barros-Filho, Leticia Helena Theodoro, Luiz Antonio Borelli Barros, Rafael Scaf de Molon

**Affiliations:** ^1^Department of Diagnosis and Surgery, São Paulo State University-UNESP, School of Dentistry, Aracatuba SP 16015-050, Brazil; ^2^Department of Dental Materials and Prosthodontics, São Paulo State University-UNESP, School of Dentistry, Aracatuba, SP 16015-050, Brazil; ^3^Department of Oral Surgery, Dental School, Araraquara University-UNIARA, Araraquara 14801-340, Brazil; ^4^Department of Social Dentistry, São Paulo State University-UNESP, School of Dentistry, Araraquara, Sao Paulo 14801-930, Brazil

## Abstract

Contemporary dentistry has increased the demand for predictable functional and esthetic results in a short period of time without compromising the long-term success of rehabilitation. Recent advances in surgical techniques have provided alternatives that allow the prosthetic rehabilitation of complex implant-supported cases through minimally invasive techniques. In this context, immediate dentoalveolar restoration (IDR) was described aiming at restoring function and esthetics through the reconstruction of lost periodontal tissues followed by immediate implant placement in order to minimize treatment time and surgical morbidity in a one-stage approach. Therefore, the aim of this clinical case is to describe the reconstruction and rehabilitation of a hopeless tooth in the maxillary region in a one-stage approach by means of IDR. The proposed steps to rehabilitate the case involved atraumatic dental extraction, immediate implant placement, and hard tissue augmentation by means of cortical-medullary bone graft harvested from the maxillary tuberosity. Afterwards, a provisional restoration was manufactured and installed to the implant allowing immediate prosthesis provisionalization and function in the same operatory time. Six months after the surgical procedure, the final prosthesis was manufactured and installed. The follow-up of nine years demonstrated the preservation of hard and soft tissue without tissue alteration and a successful esthetic outcome. The surgical protocol used allowed the ideal three-dimensional placement of the implant with the restoration of the bone buccal wall, favoring the esthetic and functional outcome of the case with harmony between white and pink esthetics. In conclusion, the employed treatment validated immediate implant-supported restoration of the missing tooth with high predictability. Furthermore, this protocol resulted in fewer surgical interventions, regeneration, and preservation of peri-implant tissues reaching the patient's expectations.

## 1. Introduction

Contemporary periodontology has presently reached such a high level of excellence that no longer allows the rehabilitation of hopeless teeth that do not effectively imitate the natural teeth and the adjacent periodontal tissues aiming at achieving the balance between esthetic and function [1]. Nowadays, the prosthetic rehabilitation should prioritize the biological focus establishing a physiological periodontal environment in close harmony with the surrounding tissues [2–4].

In order to obtain long-term functional, biological, and esthetic outcomes with immediate dental implants in fresh extraction sockets, a proper surgical and prosthetic planning should be carefully considered taking into consideration the minimal surgical invasiveness, higher outcome predictability, and decreased treatment time and morbidity to the patient. In light of the scientific improvements related to surgical techniques, biomaterials, and implant surfaces, the clinicians are now able to recommend surgical and prosthetic treatments with increased soft and bone tissue long-term stability, short treatment period, and enhanced functional and esthetic outcomes [5–7].

Scientific evidences have been demonstrated that immediate loading of implants in the esthetic region is indicated due to the partial loss of buccal bone wall in height (3.8 mm) and width (1.4 mm) 6 months after of tooth extraction [8]. The absence of buccal bone to support the gingival tissue and/or the corrected tridimensional positioning of the implant (periodontally compromised teeth) compromise the pretreatment anatomy of the alveolus, and therefore, preservation and/or reconstruction of soft and hard tissue play a pivotal role to ensure maintenance of periodontal tissues [9–11].

Immediate dentoalveolar restoration (IDR), a surgical technique first described by da Rosa et al. [12] in 2013 is designated as a one-stage approach that permits atraumatic tooth removal, instant installation of implants, reconstruction of the buccal bone wall with autogenous graft and connective tissue graft, and, finally, provisional restoration at the same operatory time [4, 11–13]. IDR has been shown to have several advantages compared to the conventional protocol as follows: (a) lower overall treatment time; (b) reduced bone resorption; (c) immediate esthetically acceptable restoration; (d) greater patient acceptance; (e) faster return of function; (f) improvements in soft tissue profile; (g) stability of the soft and bone tissues; and (h) no need for removable prosthesis [2, 5, 6, 12, 14]. On the other hand, IDR has also some drawbacks mainly related to unpredictability of site morphology, limited amount of autogenous soft and bone tissue available, and the remaining bone defect between the bone wall and the implant [15–17]. Therefore, there are some indicators that should be evaluated before the indication of IDR, such as the establishment of a precise diagnostic, sufficient amount of bone beyond the root apex to allow primary implant stability, lack of extensive gingival recession, effective application of current knowledge in new materials and techniques, and an integrative and multidisciplinary treatment plan [18].

Consequently, the aim of this report was to describe a clinical case of implant rehabilitation in a compromised tooth by means of IDR highlighting the aspects that are essential to achieve the long-term stability of periodontal tissues and implant function.

## 2. Case Description

**Figure 1:**
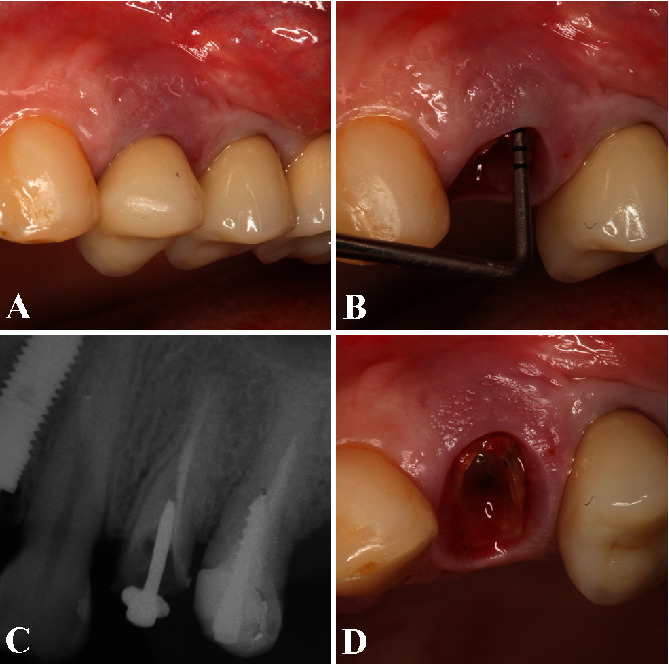
(A) The clinical examination showed class I tooth mobility, unsatisfactory endodontic treatment, and a ceramic crown. (B) The periodontal probing confirmed 10 mm of probing depth in the buccal side. (C) Periapical radiograph revealed an inadequate endodontic treatment and vertical bone loss on the mesial and distal of the tooth, and the bone height above the root apex was 8 mm. (D) After prosthesis removal, a vertical fracture in the mesial side of the teeth was observed, which indicated its extraction.

This case presents a 36-year-old female patient who sought treatment for her maxillary left first premolar in the Department of Periodontology, School of Dentistry at Aracatuba, UNESP. She presented with a ceramic crown on tooth 24, and her main complaint was excessive tooth mobility, pain during mastication, tooth fragility, and bleeding during chewing or even spontaneously bleeding. Her medical history was unremarkable, and she denied use of alcohol or smoke and medications. The clinical examination showed endodontic treatment, signs of class I tooth mobility, and a ceramic crown (Figure 1(A)). During periodontal probing, it was verified 10 mm of probing depth in the buccal side (Figure 1(B)). Her periodontal biotype was classified as thick, with a sufficient amount of keratinized gingiva. Periapical radiograph revealed an inadequate endodontic treatment, vertical bone loss on the mesial, and distal side of the tooth, and the reminiscent bone height above the root apex was 10 mm (Figure 1(C)). After removing the prosthesis, it noted a vertical fracture in the mesial side of the teeth, which indicates its extraction (Figure 1(D)). The treatment proposed to the patient was the IDR technique with simultaneous regenerative procedure, dental implant placement, and prosthesis provisionalization in a one-stage approach. The patient signed an informed consent authorizing the proposed treatment.

**Figure 2:**
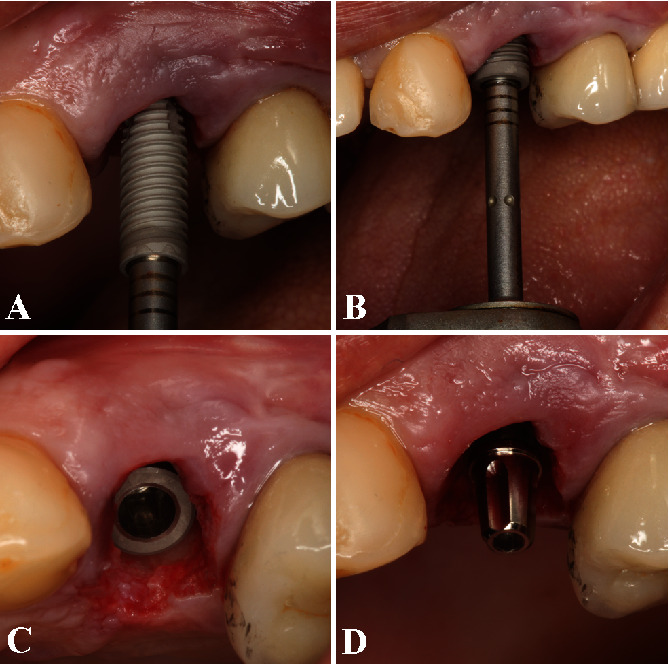
(A) After tooth removal, a dental implant (3.5 × 13 mm, Cone Morse Drive, Neodent, Curitiba, PR, Brazil) was placed into the fresh extraction socket. The drilling sequence started with a spear drill, followed by a 2 mm cylindrical drill, and a 3.5 mm conical-shaped drill concerning the mesial, distal, and palatal spaces to accomplish adequate tridimensional positioning. (B) The implant placement was performed with a flapless technique preserving the interdental papillae. (C) Implant alignment was slightly palatal, at least 2 mm distant from the nearby teeth in the mesial and distal region, and the primary implant stability achieved was 42 Ncm. (D) A titanium cylinder was attached to the implant platform.

The surgical procedure started with a local anesthesia in the buccal and in the palatine area with lidocaine 2% and epinephrine 1 : 100.000 (DFL, Rio de Janeiro, RJ, Brazil). Then, a sulcular incision was performed with a 15C blade, and the tooth was atraumatically extracted with a flapless technique using a periotome without compromising the interdental papillae. After tooth removal, the socket was curetted, and the granulation tissue was carefully removed. Next, a dental implant (3.5 × 13 mm, Cone Morse Drive, Neodent, Curitiba, PR, Brazil) was placed into the fresh socket after the drilling sequence (using a spear drill, a 2 mm cylindrical drill, and 3.5 mm conical-shaped drill) respecting the mesial, distal, and palatal distances to achieve proper tridimensional implant positioning [19]. The alignment of the implant was slightly palatal, at least 2 mm distant from the adjacent teeth (mesial and distal), and the primary implant stability of 42 Ncm was reached with a torque controller. The palatal positioning of the implant allowed the placement of an implant with an adequate diameter (3.5 mm), which favors the long-term clinical success of the case, especially when immediate implant placement is the selected approach. The apical and palatal residual alveolar bone was sufficient to provide enough primary implant stability. Afterward, a titanium cylinder was connected to the implant platform (Figures 2(A)–2(D)).

**Figure 3:**
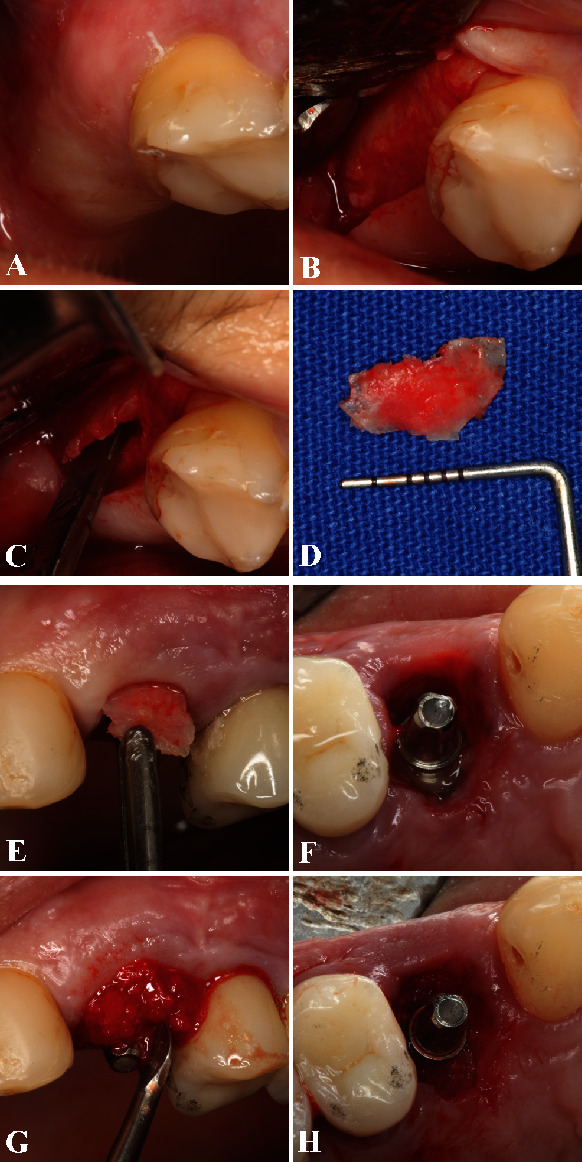
(A) The maxillary tuberosity was the region utilized to obtain the autogenous bone block graft. (B) After local infiltrative anesthesia, a vertical and crestal incision was performed following a full-thickness flap to access the right maxillary tuberosity. (C) A straight chisel (Quinelato, Rio Claro, SP, Brazil) was used to harvest the block bone. (D) The bone graft was customized according to the form of the bone defect, (E) and it was then inserted gently into the alveolar defect above the implant threads using the flapless technique. (F) Occlusal view of the fresh extraction socket with the dental implant positioned immediately after the buccal wall reconstruction with the tuberosity bone graft, allowing a reminiscent gap between the block bone and the implant. (G) The gap was filled with particulate bone harvested from the tuberosity and ground with a bone mill (Neodent, Curitiba, PR, Brazil) to guarantee the bone block maintenance and support. (H) Final occlusal view of the particulate bone completely inserted into the gap between the block bone and the implant.

Subsequently, an autogenous cortical-medullary bone block graft removed from the maxillary tuberosity was utilized to reconstruct the lost buccal bone wall. The bone graft was harvested under local infiltrative anesthesia (as described above) with a vertical and crestal incision followed by raising a full-thickness flap to access the maxillary tuberosity. A straight chisel (Quinelato, Rio Claro, SP, Brazil) was used to obtain the bone graft, and subsequently, it was custom-fitted directly into the alveolar defect above the implant according to the size and shape of the bone defect. The gap between the block bone and the implant was filled with particulate bone also collected from the tuberosity and ground with a bone mill (Neodent, Curitiba, PR, Brazil) to ensure block bone graft stabilization (Figures 3(A)–3(H)). After the surgical procedure, sutures were not necessary since there is no soft tissue incision and the particulate bone was secured with the provisional crown. It is important to mention that if we have raised a full-thickness flap, the bone graft procedure would be easier to perform. However, the long-term success of the case would be compromised due to the elevation of periosteum. Therefore, the IDR technique preconizes for atraumatic procedures to overcome such drawbacks of raising the periosteum and the detachment of interdental papillae.

**Figure 4:**
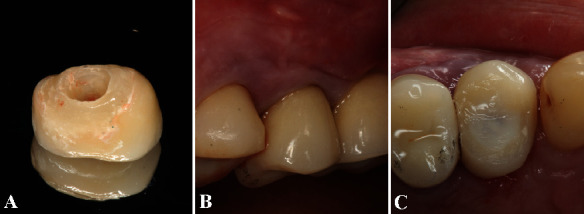
(A) After the surgical procedures, the provisional resin crown was customized. (B) Prosthesis adjustments were performed to guarantee a proper emergence profile. (C) The tooth incisal height was reduced to avoid occlusal contact during chewing and phonation.

A temporary resin crown was positioned over the titanium cylinder immediately after the bone graft procedure. Prosthesis adjustments were performed to ensure a proper emergence profile and to reduce the incision height avoiding occlusal contact. The patient received postoperative recommendations comprising oral hygiene teachings and plaque control monthly for 3 months (Figures 4(A)–4(C)).

**Figure 5:**
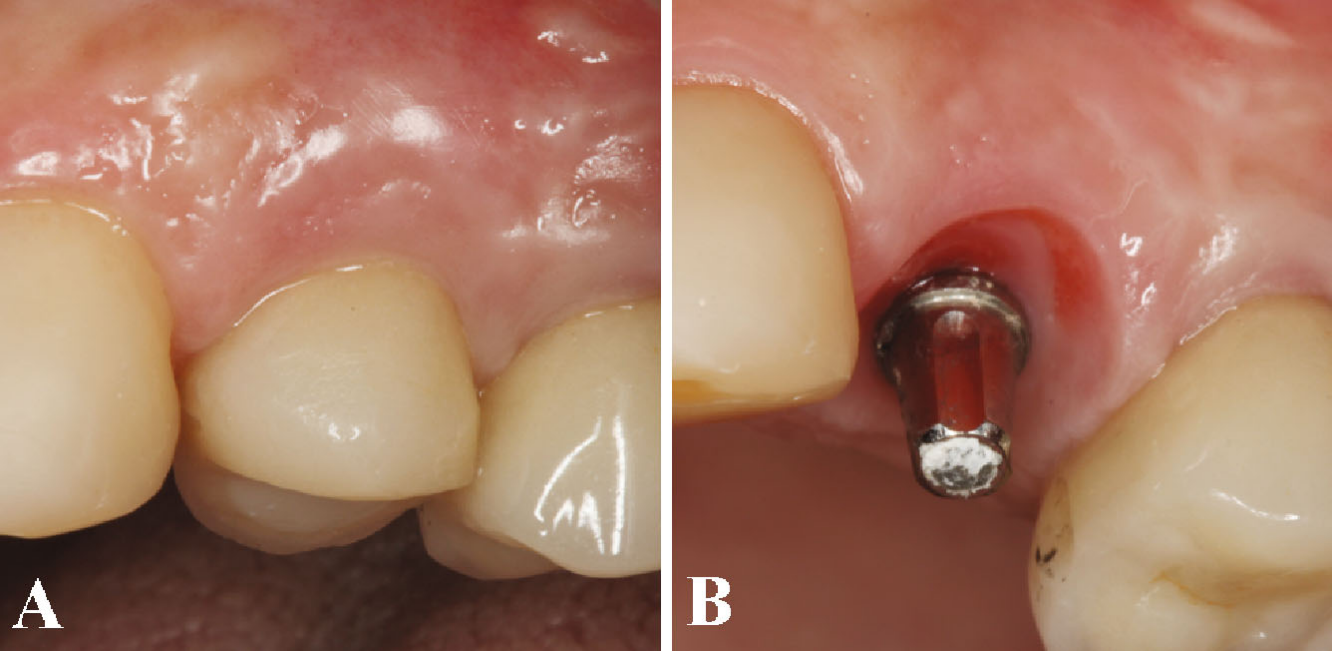
(A) Follow-up after 6 months showed a favorable esthetic result without gingival recession. (B) The clinical view after removal of the provisional crown showed excellent tissue healing and contour with no tissue inflammation.

After 6 months postoperatively, the result showed an optimal esthetic outcome without a gingival recession, probing depths, or tissue inflammation (Figures 5(A) and 5(B)).

**Figure 6:**
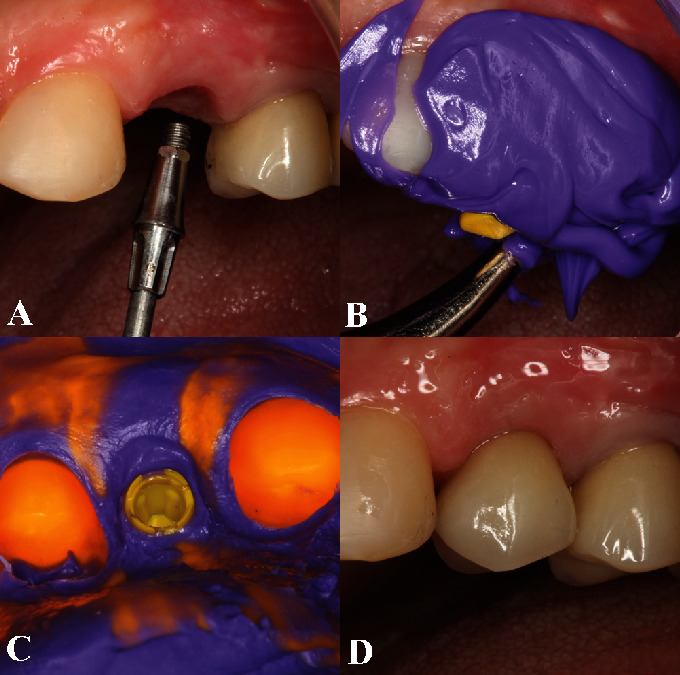
(A) A titanium abutment was installed with its cervical portion narrower than the implant diameter to create an esthetic contour of the gingival margin. (B) Insertion of light body silicone around the plastic abutment impression coping. (C) Final completed impression with the impression coping into silicone impression. (D) A porcelain crown of IPS Empress II was manufactured and cemented over the abutment. The clinical aspect demonstrated a satisfactory contour at the gingival margin.

The final prosthetic phase started with transfer impression. A stock titanium abutment was installed, in which its cervical portion is narrow than the implant width to produce an esthetic contour of the gingival tissue. Next, a definitive porcelain crown was manufactured and cemented over the abutment (Figures 6(A)–6(D)).

**Figure 7:**
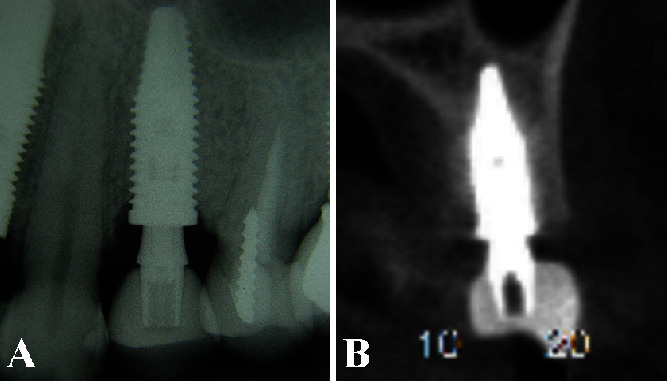
(A) The periapical radiography showed a complete implant osseointegration without signs of bone resorption. (B) The computed tomography scan showed no bone alterations in width and height surrounding the implant.

The clinical aspect demonstrated a satisfactory contour at the gingival margin. Likewise, the periapical radiographic and the computer tomography both showed a complete bone healing around the implant. Therefore, IDR treatment effectively replaces the tooth, obtaining an adequate esthetic and functional result (Figures 7(A) and 7(B)).

**Figure 8:**
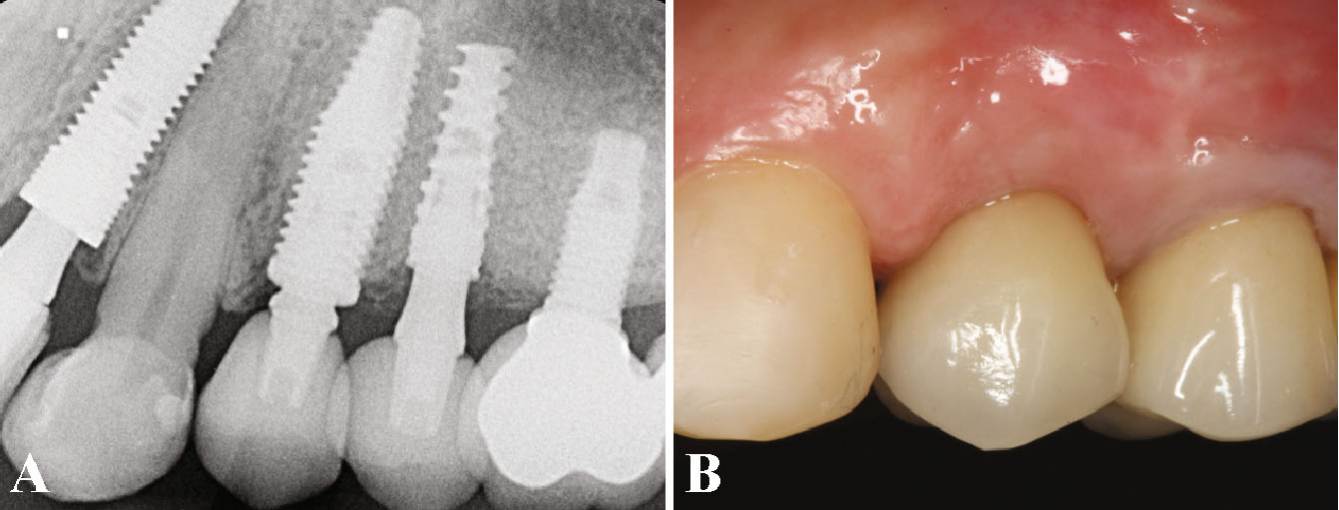
(A) Periapical radiographs after 9 years of follow-up showed the implant position and the vertical bone gain without marginal bone resorption. (B) The clinical examination revealed an adequate gingival thickness, without gingival recession or probing depths, confirming the satisfying esthetic result by the IDR treatment.

The 9 years of follow-up have shown an adequate width and thickness of gingival architecture and satisfactory esthetic result without apical gingival migration or probing depths. Moreover, periapical radiographs showed the ideal tridimensional position of the implant and the increased vertical bone gain without marginal bone loss. Thus, the functional and esthetic expectations of the patient were accomplished by the IDR technique (Figures 8(A) and 8(B)).

## 3. Discussion

Currently, dental implants placed immediately after the removal of a hopeless tooth are still a challenge for clinicians, especially while one or two bone walls are absent due to dental trauma or periodontal disease. Immediately after tooth extraction, partial reabsorption of the buccal bone wall in height and width will occur [8, 20]. In order to avoid such spontaneous remodeling of soft and hard tissues after tooth removal, the IDR technique was described by da Rosa et al. in 2013 [12], as an alternative to the use of guided bone regeneration.

To obtain successful outcomes using the IDR technique, it is necessary to perform atraumatic tooth extraction without vertical or horizontal incision (flapless surgery) to preserve the reminiscent soft and hard tissue prior to bone reconstruction and implant placement [6]. Raising a full-thickness flap in the IDR technique is not preconized since the elevation of the periosteum would probably result in bone loss in the long-term follow-up and might result in migration of the gingival margin due to soft tissue manipulation and detachment of interdental papilla. In this context, minimal trauma to the soft tissue and to the alveolar bone during the surgery plays an important role for the long-term clinical success of oral rehabilitation. Furthermore, the ideal tridimensional positioning of the implant, primary implant stability, gap filling with particulate bone, and the correct adaptation of the block bone graft outlining the defect shape are critical for the IDR success [6, 12]. Therefore, the IDR offers great benefits to patients needing immediate implant rehabilitation in compromised fresh extraction socket.

The advantages of the IDR technique could be highlighted through the case report described. Firstly, the immediate esthetical outcome and the functionalization of the prosthesis make the treatment more acceptable to the patient [1, 21]. Additionally, the surgical morbidity is similar compared to a conventional implant placement in a two-step approach (after complete healing of the soft and hard tissue after tooth extraction) [21]. More importantly, the stabilization of the gingival margin and the alveolar bone thickness and height after 9 years of follow-up were maintained over the years with the treatment employed [21]. Although the case report has resulted in adequate stabilization of the soft and hard tissue over time, a previous study indicated that instantaneous loading of titanium implants in fresh extraction sockets might not fully prevent bone changes and apical migration of the gingival tissues [22]. Therefore, all the necessary steps to complete the IDR should be strictly followed to obtain longevity of the achieved results on the periodontal tissues.

The anatomy of the maxillary bone in the anterior region is characterized by a thin bone buccal wall, which, in many instances, is reabsorbed following periodontitis or dental trauma. Thus, the implant placement should be installed in a more palatal positioning to avoid bone fenestration and to allow primary implant stability. A more palatal approach to place the implant might result in a gap between the implant and the buccal bone. Therefore, this gap should be filled with particulate bone graft (harvested from the maxillary tuberosity) to restore the contour and volume of tissue, according to the case presented favoring the vascularization and regeneration of peri-implant bone healing [23]. If the resorption of alveolar buccal bone is too severe, the IDR technique preconizes the placement of a cortical-medullary bone (collected from the tuberosity) to reconstruct the lost tissues, as described. The placement of cortical-medullary bone will result in the restoration of the supporting periodontal tissues, which will favor the long-term stability of soft and hard tissues [1].

An important consideration that should be taken into account is the gingival margin migration. Ideally, it is preconized that the thickness of keratinized gingiva should be approximately 2 mm thick to avoid gingival recession and stabilization of the margin [24, 25]. Therefore, if the gingival tissue is too thin, it is recommended to place a subepithelial connective tissue graft over the bone buccal wall to increase the periodontal protective tissues and to restrain the change of apical migration of the gingiva and to increase the final esthetic result similar to the adjacent teeth [26], as described in our case reported.

Although the IDR present innumerous advantages, some limitations of the technique should be also listed. Previous studies clarified the requirement for tooth extraction followed by immediate implant placement [19, 27]. The surgical requirements mentioned for classical IDR are primary implant stability, the presence of buccal bone, and sufficient amount of bone in an apical position to allow primary implant stability [19, 27]. However, as shown in the present case, the missed buccal bone had not limited the implant placement, and the buccal bone reconstruction was performed immediately [2, 28]. The reconstruction of the buccal bone wall using the tuberosity bone is limited to small bone defects and patients with sufficient mouth opening [29, 30]. Besides, the use of an implant with surface treatment to accelerate the osseointegration process instead of using a machined titanium implant is also recommended [31, 32].

The main prosthetic considerations in the IDR technique to obtain the long-term success consist of a correct emergence profile to avoid peri-implantitis, and, consequently, inflammatory conditions [33–37]. The principal prognostic risk for further peri-implantitis is previous periodontal disease [38–42]. In cases of tooth loss due to periodontitis, some additional cares are necessary such as oral health awareness, oral hygiene instructions, and reduced visits to the dental office for periodontal supportive care [38, 39]. Moreover, a sufficient amount of keratinized gingiva (2 mm) and the positioning of the implant platform located 3 mm apical of the cementoenamel junction of the adjacent tooth is recommended [43–45]. These special cares provide a stable biological distance in the peri-implant space, allowing bone formation on the cervical implant surface to avoid further peri-implantitis after the IDR technique [45, 46].

## 4. Conclusions

Collectively, this clinical case demonstrated the feasibility of the IDR technique to reconstruct the lost periodontal tissues followed by implant placement and functionalization. Of importance, all the described steps were conducted in a one-stage approach, which is the hallmark of the technique. The proposed treatment was able to restore the function and esthetics and fulfill the patient's expectations.

## Data Availability

The data used to support the findings of this study are available from the corresponding author upon request.
